# Postural Control Is Not Systematically Related to Reading Skills: Implications for the Assessment of Balance as a Risk Factor for Developmental Dyslexia

**DOI:** 10.1371/journal.pone.0098224

**Published:** 2014-06-03

**Authors:** Håvard Loras, Hermundur Sigmundsson, Ann-Katrin Stensdotter, Joel B. Talcott

**Affiliations:** 1 Sør-Trøndelag University College, Trondheim, Norway; 2 Norwegian University of Science and Technology, Trondheim, Norway; 3 Aston Brain Centre, School of Life and Health Sciences, Aston University, Birmingham, United Kingdom; Birkbeck College, United Kingdom

## Abstract

Impaired postural control has been associated with poor reading skills, as well as with lower performance on measures of attention and motor control variables that frequently co-occur with reading difficulties. Measures of balance and motor control have been incorporated into several screening batteries for developmental dyslexia, but it is unclear whether the relationship between such skills and reading manifests as a behavioural continuum across the range of abilities or is restricted to groups of individuals with specific disorder phenotypes. Here were obtained measures of postural control alongside measures of reading, attention and general cognitive skills in a large sample of young adults (*n* = 100). Postural control was assessed using centre of pressure (CoP) measurements, obtained over 5 different task conditions. Our results indicate an absence of strong statistical relationships between balance measures with either reading, cognitive or attention measures across the sample as a whole.

## Introduction

Developmental disorders are typically defined when a child experiences failure in attaining age-appropriate levels of achievement in one or more specific cognitive or behavioural domains. These deficits in achievement occur in the absence of other obvious endogenous or exogenous causes, which results in diagnoses weighted heavily by exclusionary compared to inclusionary criteria.

Reading disorder, or developmental dyslexia, is defined by achievement of reading skills that are considerably lower than would be expected from the profile of measured abilities in other cognitive domains, when neurological and sensory function is normal, and other socio-cultural factors, including access to education, are at least adequate [1]. Dyslexia is highly heritable; approximately half of the population variance in reading skills and deficits thereof can be attributed to genetic factors [2]–[4]. These risks explain a similar proportion of phenotypic variance, irrespective of whether reading is assessed with a continuous measure or as a categorical phenotype, suggesting that the same genes impact upon common cognitive skills that constrain reading achievement in both normal and atypical development [4], [5].

Deficits in the representation and cognitive processing of phonological information are recognized as core risk factors for the genesis of poor reading ability across the lifespan [6], [7]. In preliterate children, phonological awareness skills strongly predict future reading skill across a broad age and ability range [8]. Despite the prominence of phonological deficits as a potent risk factor [6]–[9], the phenotype of dyslexia often encompasses a broad constellation of information processing deficits that extend well-beyond the phonological domain [10]–[12]. One of these areas is in motor control, where previous studies have reported difficulties in measures of balance and posture that are associated both with dyslexia [13]–[18] and with reading skills across the range of abilities in the general population [19]. Nicolson, Fawcett & Dean [20] proposed that the apparent link between balance and reading in dyslexia may result from mild impairment of the development and functioning of the cerebellum. This hypothesis suggests that cerebellar dysfunction in dyslexia impairs not only motor control and coordination, but also limits the extent to which cognitive skills such as reading can be learned and automated. Several empirical studies of dyslexia have reported differences in cerebellar morphology [21] and neurochemistry [22] consistent with this hypothesis. Some current neuropsychological screenings for dyslexia risk incorporate measures of balance or of other motor skills as subscales for the prospective assessment of disability risk [23], [24].

An important consideration in the evaluation of measures of posture and balance for the assessment of *specific* risk for dyslexia, however, centres on the ability of such assessments to discriminate between individuals at risk for reading difficulties from those without impairments and those with other developmental disorders. Deficits in motor control may also be associated with other disorder phenotypes, such as ADHD [25] and developmental coordination disorder (DCD) [26], both of which have particularly high diagnostic co-morbidity with dyslexia. The overlap between these disorders is high enough to suggest that they share underlying risk factors, yet a demonstration of the positive predictive value of deficits in posture and balance for ascribing specific risk for dyslexia relies on the demonstration of co-variance between motor function and the core symptom of dyslexia, namely poor reading skill. The research in this area is equivocal; few studies have sufficiently examined these relationships with continuous variables of reading and posture in typically developing readers, particularly with measures that are both objective and sufficiently sensitive (cf., [16], [18], [27]–[29]).

In a series of case-control studies, Fawcett, Nicolson and colleagues [30], [31] reported that difficulties in motor skills, and particularly measures of balance, provided high diagnostic sensitivity for dyslexia. They demonstrated that the majority of adults with dyslexia but only a small proportion of the control group showed at risk performance on a balance measure [30]. In children, similar discriminative validity was reported [31]. Several other studies have reported contrasting findings, however. Ramus et al. [27] reported that only about a quarter of adults with dyslexia could be described as having abnormal balance performance. Wimmer, Mayringer and Rayberger [32] also presented negative evidence and suggested that the presence of balance deficits in dyslexia may be better explained by the effects of an often unmeasured third variable, namely the presence of co-morbid developmental disorders, and specifically ADHD.

In an attempt to reconcile this apparently conflicting literature, Rochelle and Talcott [33] conducted a meta-analysis to quantitatively assess the evidence for a balance deficit in dyslexia. Effect-size estimates for balance measures, obtained from 15 case-control studies, revealed overall strong effects between groups (*d* = .64) but with highly inhomogeneous effect-sizes across studies. Moderator variable analyses suggested that the variability in effect-sizes in the population of studies was strongly modulated (r = ∼0.8) by the probable presence of co-morbid but often unmeasured ADHD symptoms in the samples, but much less so with variability in measures of reading and component skills. In a follow-up study Rochelle, Witton & Talcott [29] experimentally replicated this effect, showing that balance measures co-varied substantially with ADHD symptoms, but not with measures of reading skill, in a between-group comparison of good and poor adult readers.

In this study, we investigated the association between measures of postural control, reading, attention, and cognitive ability in a large opportunity sample of young adults. We obtained measurements of centre of pressure (CoP) for 5 different standing balance conditions, using a force plate to acquire postural displacements in real time. CoP measures of postural sway provide sensitive and objective assessment of balance and were used as predictors of the reading and cognitive constructs that comprise the core symptom dimensions in dyslexia.

## Materials and Methods

### Participants

The experimental procedures were initiated following approval of the protocol by the central regional ethics committee for medical research (REC Central). All subjects provided written consent prior to participating in the study and all procedures were carried out in accordance with the code of Ethics of the World Medical Association (Declaration of Helsinki).

The participants (*n* = 100), including 37 men and 63 women, were recruited from a college community in Norway. All were neurologically healthy adults, with a mean age of 22.6 years (SD 2.6) across the entire sample.

In their meta-analysis, Rochelle and Talcott [33] reported average correlations between balance and reading skill of .31 and a mean *d* effect size of .64 (*r* effect-size equivalent of .41) for between-group comparisons of groups with and without dyslexia. Using these effect-size estimates, the statistical power of this study (*n* = 100) is in excess of 80% for detection of correlations at .3 and above and in excess of 90% for correlations .4 and higher.

### Stimuli

#### Cognitive measures

Participants were assessed on a battery of psychometric constructs, including the Digit Symbol-Coding and Symbol Search subtests of the Wechsler Adult Intelligence scales [34], and non-verbal reasoning using the Raven's progressive matrices [35]. We also administered the Rapid Naming measure from the Dyslexia Adult Screening Test (DAST) [24]. Raw scores on all the cognitive measures were obtained as measures of individual performance for use in subsequent statistical analyses.

Participants also completed a self-report measure of ADHD-symptoms: the World Health Organization adult self-report scale (ASRS 1.1) [36]. The ASRS includes 18 questions about the frequency of DSM-IV symptoms of adult ADHD over the past 6 months, using a 5-point Likert scale. The individual total score obtained across all 18 questions was used for further analysis.

#### Reading skill

Reading achievement was measured using *Wordchains* [37], [38], a measure of fluency of word recognition. Wordchains provides a reliable and valid test of the speed and accuracy of word recognition skills across a large age range [37]–[39]. It avoids ceiling effects associated with measures based on accuracy only, which makes it particularly well-suited for the assessment of reading achievement in languages with more transparent orthographies such as Norwegian. This task has been validated against reading outcomes in both English and Scandinavian languages [39], with scores correlating highly with concurrent measures of reading skill [37], [38] across a broad age and ability range.

Participants were given a booklet containing rows of Norwegian words presented in the form of 90 ‘chains’ (for e.g., presentedformchains) and were given 4 minutes to divide as many chains as possible into their component words by drawing a line to designate the appropriate word boundaries (i.e., presented/form/chains). The number of correctly segmented words in the time allowed is adopted as the operational definition of performance. For the age of the adult participants in our sample, a raw score of 34 or lower corresponds to a standard score 1sd below the standardized population mean. Descriptive statistics for the participant samples on the test battery are shown in Table 1.

**Table 1:** Descriptive statistics for the participant sample (*n* = 100) on the study measures.

Variable (unit)	Mean (SD)	Min-Max
Age (years)	22.6 (2.6)	19–33
Ravens Matrices (raw score)	52.4 (4.5)	38–60
Symbol Search (raw score)	39.1 (6.3)	27–57
Digit Symbol (raw score)	88.2 (13.6)	52–126
Rapid Naming (s)	26.0 (4.7)	18.7–43.6
WordChains (raw score)	59.1 (11.0)	34–90
ASRS (score)	28.6 (6.9)	16–60
Normal Standing ML	1.46 (.66)	.52–3.99
AP	4.36 (1.65)	1.89–10.68
Feet Together ML	5.09 (1.26)	2.85–9.64
AP	5.62 (2.09)	2.44–12.82
Semi-tandem ML	6.15 (1.23)	3.18–10.22
AP	5.04 (1.64)	2.45–10.47
Tandem ML	6.90 (1.35)	4.10–13.04
AP	7.25 (3.97)	2.55–23.11
Right ML	7.91 (3.54)	4.73–26.85
AP	9.13 (3.97)	4.82–29.87
Left ML	7.50 (3.07)	4.05–23.92
AP	8.84 (2.77)	3.61–21.18

Postural displacements during the recording epochs are expressed in standard deviation units of displacement in millimetres for the centre of pressure in the medio-lateral (ML) and anterior-posterior (AP) axes. s: seconds.

#### Postural stability

CoP across the anterior-posterior (AP) and medio-lateral (ML) planes was collected with a portable force platform (Good Balance, Metitur Ltd., Finland), equipped with a strain-gauge force-transducer in each corner. The platform was connected to a three-channel, direct-current amplifier and a 12 byte AD converter, linked via a Blue-tooth connection to a stationary computer where data were collected at a sampling rate of 200 Hz and stored with proprietary software. The platform was calibrated prior to each test session, including level positioning of the unit on the floor.

**Figure 1:**
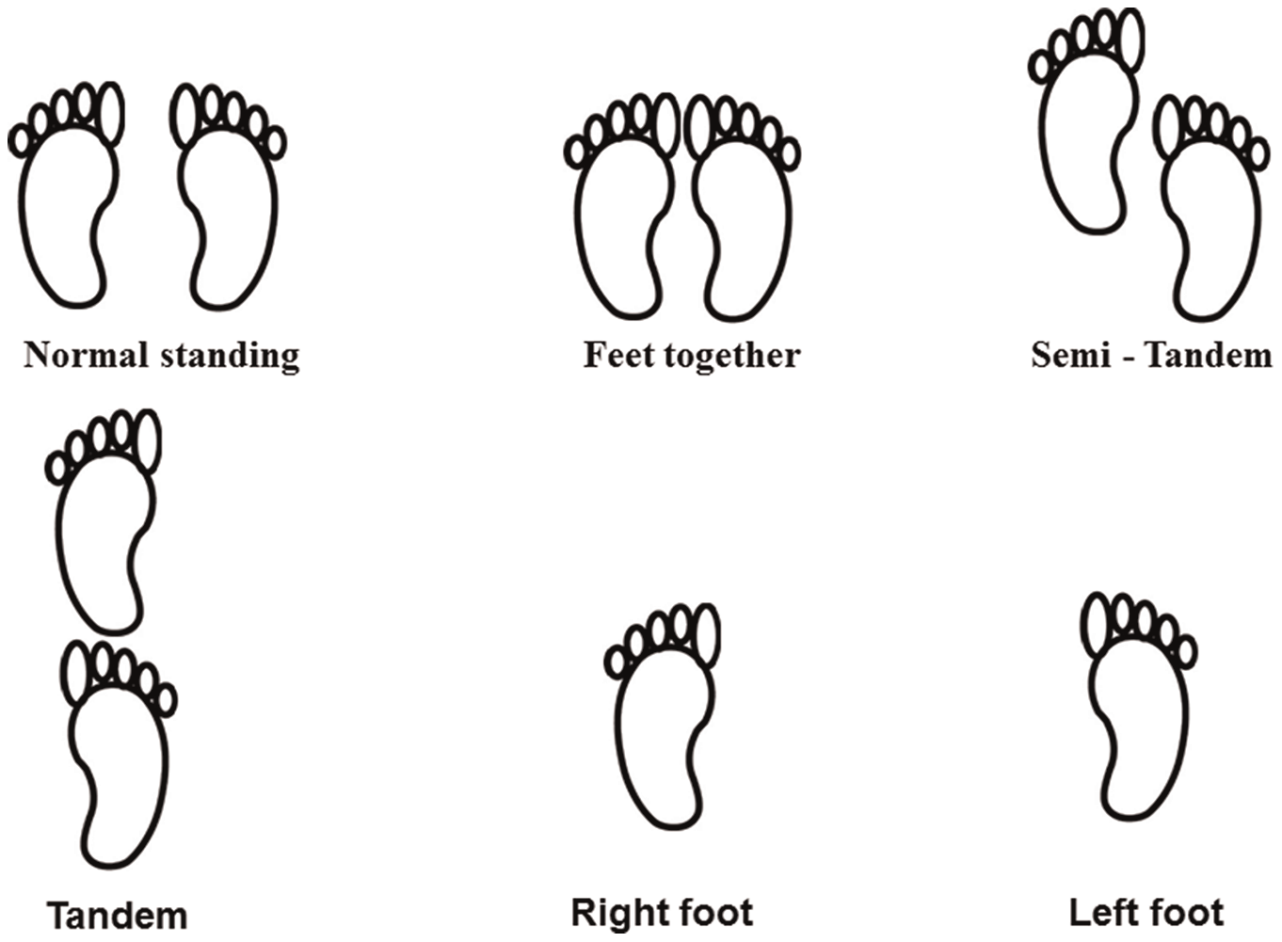
Schematic diagram of the postural control conditions employed in this study.

Static postural stability in 5 different quiet standing conditions was collected during separate one minute recording epochs [40], [41]. Figure 1 provides a visual schematic of the balance conditions completed by participants. These included: (1) *Normal standing*- the participant's normal stance, including self-chosen angle of foot position and distance between the feet; (2) *Feet together-* the participant was instructed to place their feet closely together toe-to-toe; (3) *Semi-tandem*- the subject was instructed to place the heel of one foot alongside the big toe of the opposite foot; (4) *Tandem*- the heel of one foot was placed directly in front of the other with the big toe touching the heel of the forward foot; (5) *Stork stand*- the participants stood on either the right or left foot with the sole of the other foot against the side of the supporting knee. All standing conditions were performed without shoes, with arms folded across the chest and with eyes open. Participants were instructed to step onto the force platform and to remain still and relaxed in the given stance. After finding a comfortable position, the participant was asked to fix their gaze on a point placed on a wall 4m in front of them throughout the duration of each recording epoch.

### Data analysis

Analyses of postural stability data were conducted in Matlab 7.12 (Mathworks, USA) with scripts provided by Duarte & Freitas [42]. Raw signals containing CoP displacements were filtered with a low pass, 10 Hz, second order, zero-phase Butterworth filter and detrended. Consistent with previous research (for e.g., [29]), we calculated the standard deviation of the ML and AP CoP displacements as dependent measures of postural sway.

## Results

As shown in Table 2, zero-order, parametric correlations between postural sway and cognitive variables did not exceed r = .23 (*p* = .024; Ravens and AP sway in the Tandem condition), and there were no significant relationships (maximum *r* = .16, *p*>.05) between postural sway and the WordChains or RAN measures. These data provide no evidence of any consistent pattern of association between reading and postural control variables.

**Table 2:** Zero-order Pearson product-moment correlation coefficients between cognitive and postural sway measures.

	1	2	3	4	5	6	7	8	9	10	11	12	13	14	15	16	17	18
1. Ravens		**.20**	**.20**	.02	.09	.07	.05	.13	.05	.05	.14	.01	.02	**−.23**	.09	.10	.08	.01
2. SS			**.51**	**−.28**	**.50**	.05	.04	.04	.09	.08	.01	.03	.05	.03	.01	.06	**−.22**	.09
3. DSC				**−.41**	**.55**	.08	.05	.13	.05	.03	**−.23**	.01	.15	.02	.15	.09	.09	.04
4. RSN					**−.31**	.06	.16	.05	.10	.03	.04	.03	.11	.01	.02	.10	.12	.01
5. WC						.01	.06	.06	.02	.04	.04	.03	.05	.11	.04	.01	.07	.01
6. ASRS							**.21**	.08	.07	.12	**.24**	.13	−.07	**−.20**	−.12	**−.21**	.12	.06
7. Normal Standing ML								**.60**	**.39**	**.29**	.16	**.27**	.18	.07	.02	.02	**.20**	.18
8. AP									**.26**	**.41**	.19	**.36**	.12	**.24**	**.07**	.08	.05	.15
9. Feet Together ML										**.54**	**.39**	**.34**	**.27**	**.29**	.19	.13	**.38**	**.35**
10. AP											**.33**	**.48**	**.24**	**.42**	**.15**	.13	**.32**	**.46**
11. Semi-tandem ML												**.28**	**.44**	**.23**	.21	**.28**	**.33**	**.28**
12. AP													**.28**	**.28**	.16	.15	**.24**	**.33**
13. Tandem ML														**.48**	**.35**	**.43**	**.45**	**.43**
14. AP															.15	**.25**	**.32**	**.26**
15. Right ML																**.85**	**.27**	.17
16. AP																	.19	.19
17. Left ML																		**.69**
18. AP																		

Significant correlations in bold (*p*<.05, not corrected). SS: Symbol search; DSC: Digit symbol coding; RSN: Rapid serial naming; WC: Word chains; ASRS: Adult ADHD Self Report Scale; ML: Medio-lateral; AP: Anterior-posterior.

The significant correlations observed between postural sway variables (see Table 2), coupled with inspection of anti-image matrices, Kaiser-Meyer-Olkin measures of sampling adequacy exceeding .6 and a significant Bartlett's test of sphericity (p<0.001), indicated that the covariance within ML or AP sway measures were factorable. Principal components analyses for the sway measures in each plane resulted in similar one-factor solutions, supported by inspection of scree plots and the presence of a single eigenvalues exceeding 1. Monte Carlo simulation (6 variables, 100 subjects, 1000 replications) confirmed that only these single factors in each solution had eigenvalues larger than would be expected for randomly generated data. The factor loadings of all the tasks, eigenvalues and total explained variances for the one-factor PCAs of postural sway measures are presented in Table 3. The one-factor solutions for each movement plane had moderate to relatively high loadings from all posture tasks (range .37–.81), which accounted for 41% of the total explained variance in each model.

**Table 3:** Results of the principal-component analysis of postural sway measures.

	Medio-lateral	Anterior-posterior
Normal Standing	.45	.59
Feet together	.70	.81
Semi-tandem	.69	.71
Tandem	.73	.64
Right	.50	.37
Left	.72	.64
Eigenvalue	2.46	2.47
Explained variance (%)	41.1	41.1

Factor loadings for each postural sway variable, including eigenvalues, and variance explained for the one-factor principal component solutions across each measurement plane.

Table 4 shows the correlations between the cognitive variables and the principal component of postural sway for each measurement plane. Consistent with the pattern of zero-order correlations presented in Table 2, there were neither significant nor strong statistical relationships between postural sway on the ML or AP axes and any of the cognitive or reading measures.

**Table 4:** Pearson product-moment correlation coefficients between cognitive measures and principal components of postural sway measures.

	1	2	3	4	5	6	7	8
1. Ravens		**.20**	**.20**	.02	.09	.07	−.09	−.12
2. SS			**.51**	**−.28**	**.50**	.05	−.07	−.05
3. DSC				**−.41**	**.55**	.08	−.17	.03
4. RSN					**−.31**	.06	.14	−.02
5. WC						.01	−.03	−.03
6. ASRS							.18	.03
7. ML PC								**.68**
8. AP PC								

Significant correlations in bold (*p*<.05, not corrected). Abbreviations as for Table 2, except PC (principal component).

A plausible alternative explanation for the lack of statistical relationship between postural stability variables and cognitive or reading skills across the range of participant abilities is that deficits in posture are restricted to persons at the lower end of the performance continua on these measures. As a test of this hypothesis, we selected the individuals in the sample who scored at or below the 10^th^ percentile on the WordChains (*n* = 10), ASRS (*n* = 10) or Raven's matrices (*n* = 14) and compared them to the remainder of the sample on the postural stability measures using non-parametric Mann-Whitney tests. There were no significant between-group differences on any postural control variable for the reading (WordChains, minimum *p* = .063) or non-verbal reasoning groups (Raven's, minimum *p* = .060). For the attention (ASRS) group, one significant between group effect was found for the semi-tandem task in the ML plane [*p = .022*]. The lack of consistent significant relationships between controls and extreme groups derived from the cognitive and reading measures was upheld when the same analyses were run for the principal components of postural sway across the two movement planes (minimum *p* = .290 for comparison of the attention groups on ML sway).

As a final test, we assessed whether a group with multiple instances of low scores on the reading, attention and non-verbal reasoning variables experienced postural control differences compared to controls. We identified individuals in the sample who had performance on more than one of the measures that fell below the 10^th^ percentile. Non-parametric comparisons between groups with either multiple instances of low scores, one low score, or no such scores, were not significant across the different postural control conditions for either of the two planes of displacement. This lack of statistically significant relationship was repeated when the reduced principal component measures were used as the dependent measures.

## Discussion

Postural control variables have been employed in test batteries for the assessment of specific risk of dyslexia. However, it remains unclear to what extent inter-subject variability on such measures captures individual differences in reading achievement, the primary diagnostic symptom of developmental dyslexia. Here we obtained measures of postural stability in 5 different experimental conditions, in parallel with psychometric measures of reading skill and other cognitive dimensions (e.g., ADHD symptoms) that may also statistically co-vary with motor control variables. Our data do not support the contention that measures of balance and reading skill are tightly correlated, at least in this non-clinical sample of young adults. Therefore, if reading impairment is the primary symptom upon which differential diagnosis of developmental dyslexia is to be made, then our results suggest that balance measures do not alone provide sensitivity sufficient for the assessment of specific dyslexia risk. Although our data do not support the existence of a substantial covarying relationship between reading and postural control variables, there are at least four alternative hypotheses that might explain both our pattern of results and the variability in the findings reported across previous studies in this area.

First, variability in posture and balance may only predict reading skills in children where performance in these domains has not yet reached asymptote via maturation or other developmental influences. Although our data do not address this alternative hypothesis directly, this explanation is in our view the least likely to adequately explain the inconsistency of findings for measures of balance and posture in dyslexia. Although, there are comparatively fewer studies of adults in the literature, the magnitude of between-group effect sizes for these studies are of similar magnitude to those found in studies of children [33]. Variability in the age of both the clinical and control participants was investigated as a moderating variable in the meta-analysis of Rochelle & Talcott [33], which showed that the age of participants was not a strong predictor of between-group effect sizes on balance measures.

Second, several studies have suggested that the presence of a balance deficit depends upon the paradigm used for its assessment, with difficult and dual-task paradigms more likely to yield significant group effects [43]–[45]. Larger between-group differences may result from the application of more difficult and complex paradigms, for example those involving sensory modulation [13], perturbation of the consistency of the standing surface [16], or the presence of a secondary, distractor task [44]. Dual task environments in particular have been argued to provide superior paradigms for measuring the ability to automate motor tasks, because the presence of the secondary task requires reallocation of attention resources away from the primary task (for e.g., postural control), resulting in performance decrements and thereby mitigating against ceiling performance. The effectiveness of dual tasks in this context has been interpreted by Nicolson and Fawcett [20] as evidence for impaired ‘automaticity’ of motor control in dyslexia. However, as Wimmer, Mayringer & Rayberger [32] argued, the impairment of performance in dual task conditions by participants with dyslexia may also result from the presence of ADHD symptoms such as inattention, given the additional load on attentional modulation in multiple task paradigms. We did not identify robust correlations between a self-report measure of ADHD symptoms and postural stability obtained for simple (i.e., not dual task) balance tasks in our non-clinical sample of adults. Nevertheless, previous studies have shown that the effects of task difficulty may interact with other participant attributes other than reading skill to impact upon the dependent measures of balance, for example if participants are selected for differences in attentional control variables [46], or where there are asymmetries of variability in attention skills within and/or between groups [15], [47]. The assessment of co-occurring symptoms associated with other developmental disorders may be particularly important in clinical samples where the incidence of disorder comorbidity would be predicted to be much higher than in the general population.

Rochelle and Talcott [33] identified that systematic differences in task parameters did not account for significant variance in the magnitude of between-group effects for balance. Although the presence or absence of a dual task does not, therefore, appear to be a strong predictor of effect size in studies of dyslexia, one potential contributing factor to inter-study variability could be the sensitivity of the paradigm for measuring balance and particularly the extent to which the data are obtained through purely objective empirical measures. Measurements of fine grained and often subtle individual differences in adjustments of posture are afforded by technological developments in real-time motion detection. Yet, for pragmatic reasons, the measures of balance developed for use in clinical contexts, such as those embedded in current dyslexia screening batteries [23], [24] are limited in their ability to provide precise and objective assessment of balance function. While more subjective measures of balance assessment may yield particularly large effect sizes (see for e.g., [30], [31]) several previous studies have examined balance in dyslexia using objective, experimental paradigms with fine-grained sensitivity to detect subtle differences in postural control, with some reporting moderate positive effects [16], [18], [28], and others demonstrating an absence of significant differences between groups [27].

Third, it might be argued that balance and reading skills do not correlate in the population overall, but have a relationship that occurs only in individuals with the most severely impaired reading skills. Although it should be noted that none of our sample of young adults achieved a score greater than one standard deviation below the population norm on the reading measure, when our sample was stratified to investigate the hypothesis that balance deficits are only identified in individuals in the comparative tails of the sampling distributions, we found no significant between-group effects. Recent analyses, both of the behavioural phenotype of dyslexia and of reading disability genetics [48], support the hypothesis that dyslexia is best represented by the lower tail of the normal distribution of reading skills, rather than a discontinuity or qualitatively different syndrome. Although we did not obtain a clinical sample of individuals with dyslexia, the variability on the WordChains measure (see Table 1) suggests a wide-range of abilities that would be sensitive enough to reveal correlations between reading and postural control variables if they existed in the population.

Finally, balance may not be a specific risk factor for developmental reading disability but instead comprises a more general risk factor for delayed or atypical development in domains independent from reading [28], [29], [49]. Consistent with this hypothesis, Viholainen et al [28] showed that balance was poorly correlated with reading outcomes in a large sample of children with and without family risk for dyslexia. However, they also demonstrated differences in postural control variables between the at-risk and non-risk groups, suggesting that balance was related to dyslexia, but through third variables other than reading skill. Models of disorder co-morbidity increasingly point to the idea that developmental disorders are best represented by variability across multiple performance continua rather than manifesting as discrete, categorical phenotypes. Plomin & Kovacs [50] as well as other authors (for e.g., [48], [49]) have suggested that the genetic risk factors for putatively different behavioural phenotypes are highly overlapping, and that the candidate genes are the same as those that mediate population variability on the same cognitive and behavioural skills. Diagnoses of the most common developmental disorders overlap at such a high rate that they almost certainly share underlying risk factors. Aside from dyslexia, balance deficits have been shown to occur in mathematics disorder [51], ADHD [25] and developmental coordination disorder (DCD) [52], (cf. [53]). All of these disorders diagnostically overlap with dyslexia by up to 50%, which is much higher than would be expected by the random co-occurrence of independent diagnostic entities with moderate prevalence rates (∼5%) in the population.

Our results suggest that balance difficulties are not correlated directly with the primary symptom of dyslexia, namely reading skill. The absence of significant covariance with reading skill, but in the presence of the apparently higher incidence of deficits of postural control in dyslexia [33], is consistent with the alternative hypothesis that such symptoms may comprise part of a broader set of non-specific risk factors. Such non-specific deficits might be considered overlapping, bridge symptoms (for e.g., see 54) that provide links to identify co-morbid aspects of developmental disorders rather than to specific symptom features of any one diagnostic category. Alternatively, the link between dyslexia and deficits in postural control in some individuals may be explained by the presence of disorder comorbidity [32], [33], [51], [52], with balance deficits more tightly associated with the co-occurring condition than with dyslexia.

## Conclusions

Developmental dyslexia has been previously associated with deficits in balance and postural stability, but this relationship does not hold in a putatively normal population for the prediction of reading skills. The link between motor control and reading disability is therefore almost certainly accounted for by associations with processing domains other than reading, including those in which deficits are considered symptoms of other developmental disorders. Given the high diagnostic overlap between developmental disorders, symptoms of impairment in motor control may be better accounted for as non-specific symptoms that represent the overlapping dimensions of disability risk. Instead of using motor difficulties to promote diagnosis of dyslexia specifically, the appearance of such symptoms may provide indicators that additional assessment is needed in other cognitive and physiological domains.
